# An introduction to digital determinants of health

**DOI:** 10.1371/journal.pdig.0000346

**Published:** 2024-01-04

**Authors:** Swathikan Chidambaram, Bhav Jain, Urvish Jain, Rogers Mwavu, Rama Baru, Beena Thomas, Felix Greaves, Shruti Jayakumar, Pankaj Jain, Marina Rojo, Marina Ridao Battaglino, John G. Meara, Viknesh Sounderajah, Leo Anthony Celi, Ara Darzi

**Affiliations:** 1 Department of Surgery & Cancer, Imperial College London, St. Mary’s Hospital, London, United Kingdom; 2 Institute of Global Health Innovation, Imperial College London, South Kensington Campus, London, United Kingdom; 3 Massachusetts Institute of Technology, Cambridge, Massachusetts, United States of America; 4 Dietrich School of Arts and Sciences, School of Medicine, University of Pittsburgh, Pittsburgh, Pennsylvania, United States of America; 5 Mbarara University of Science & Technology, Uganda; 6 Centre of Social Medicine and Community Health, Jawaharlal Nehru University, New Delhi, India; 7 Indian Council of Medical Research, National Institute for Research in Tuberculosis, Chennai, India; 8 Science, Evidence and Analytics, National Institute for Health and Care Excellence, England, United Kingdom; 9 Faculty of Medicine, School of Public Health, Imperial College London, United Kingdom; 10 Health Plan Consumer and Provider Technology, Highmark Health, Pittsburgh, Pennsylvania, United States of America; 11 Department of Marketing, Indiana University of Pennsylvania, Indiana, Pennsylvania, United States of America; 12 Public Health Innovation Lab, Med School, Buenos AIres University, Argentina; 13 Department of Plastic and Oral Surgery, Longwood Avenue, Boston, Massachusetts, United States of America; 14 Division of Pulmonary, Critical Care and Pain Medicine, Beth Israel Deaconess Medical Center, Boston, Massachusetts, United States of America; 15 Laboratory for Computational Physiology, Institute for Medical Engineering and Science, Massachusetts Institute of Technology, Boston, Massachusetts, United States of America; The University of Melbourne, AUSTRALIA

## Abstract

In recent years, technology has been increasingly incorporated within healthcare for the provision of safe and efficient delivery of services. Although this can be attributed to the benefits that can be harnessed, digital technology has the potential to exacerbate and reinforce preexisting health disparities. Previous work has highlighted how sociodemographic, economic, and political factors affect individuals’ interactions with digital health systems and are termed social determinants of health [SDOH]. But, there is a paucity of literature addressing how the intrinsic design, implementation, and use of technology interact with SDOH to influence health outcomes. Such interactions are termed digital determinants of health [DDOH]. This paper will, for the first time, propose a definition of DDOH and provide a conceptual model characterizing its influence on healthcare outcomes. Specifically, DDOH is implicit in the design of artificial intelligence systems, mobile phone applications, telemedicine, digital health literacy [DHL], and other forms of digital technology. A better appreciation of DDOH by the various stakeholders at the individual and societal levels can be channeled towards policies that are more digitally inclusive. In tandem with ongoing work to minimize the digital divide caused by existing SDOH, further work is necessary to recognize digital determinants as an important and distinct entity.

## Introduction: What is digital health, and why is it important?

Digital health [DH] refers to the use of technology to deliver healthcare services [1]. The American Medical Association [AMA] defines it as digital platforms and solutions that engage consumers for health and wellness purposes, collect and use their clinical data, and manage health outcomes and quality of care [2,3]. Broadly, it includes categories such as mobile health, health information technology, wearable devices, health and wellness online platforms and digital equipment, telehealth and telemedicine, personalized medicine, and artificial intelligence [AI] tools [4,5]. In recent years, the incorporation of technology in these forms within healthcare has increased in both developed and developing countries, marked by the acknowledgement of digital health as a vital component of planning and providing healthcare services by organizations and governments. For example, the 2019 World Health Organization [WHO] global strategy report on digital health established the priority of the digital health strategy and put forward guiding principles, strategic objectives, action framework, and implementation plans to promote the development of global digital health and to achieve universal health coverage and health-related sustainable development goals [6].

Within most Organisation for Economic Co-operation and Development [OECD] countries, specific organizations such as the Australian Digital Health Agency have been tasked to implement the use of digital health [7]. Similarly, in India, the Ayushman Bharat Digital Mission [ABDM] has been created support the integrated digital health infrastructure of the country in line with other digital government programs such as the Aadhaar identification program [1]. While digital health confers several benefits and is clearly the way forward, there is also considerable evidence demonstrating that it can introduce and exacerbate the existing social disparities [8]. These disparities need to be contextualized alongside the social determinants of health [SDOH] to help understand the general disparities in universal healthcare. These could include how sociodemographic, economic, and political factors affect individuals’ interactions with digital health systems or solutions [9]. However, within these interactions, the technology is seldom questioned and critiqued for promoting such health inequities [10]. This understanding is required to ensure that digital technology addresses these inequities in order that the right interventions are in place if digital health is to be promoted for better and accessible healthcare. In this paper, we aim to introduce the concept of digital determinants of health [DDOH], what it comprises of, and how it shapes the experiences of the individuals and social groups. We will summarize the impact of DDOH on health inequities, provide introductory examples of these, and link them to other papers within this series.

In 2000, the Millennium Development Goals acknowledged the potential role for technology in elevating the standard of healthcare services globally [11]. Fifteen years later, the rapid evolution in the functionality of digital technology and its unprecedented uptake by the world population has transformed it to become the central tenet of healthcare, as part of the Sustainable Development Goals to provide universal health coverage [12,13]. This concept was adopted for better tuberculosis [TB] control with mobile technology to promote better TB treatment adherence and provide care to the hard to reach populations [14–16]. Furthermore, the Coronavirus Disease 2019 (COVID-19) pandemic witnessed an exponential rise in digital health adoption as it became the primary channel of care delivery during the 2020 to 2021 to address challenges of social distancing and lockdown mandates in major parts of the world [17]. Taken together, while the benefits of digital technology are well-known, it is imperative to understand these digital determinants to ensure that its benefits are fully realized in the most fair and equitable way possible.

## Digital determinants of health: What do we know?

Digital determinants of health, is a novel, contemporary, and relevant construct, given its significant impact in achieving health equity. In the 2020 Lancet and Financial Times Commission report, the panel alludes to factors that drive and determine the digital transformation of healthcare [18]. In the editorial borne out of the commission, the authors highlight digital technologies as a “new determinant of health” in the title [10,19]. Following this, the WHO acknowledges the term “digital determinants of health” with example of “literacy in information and communication technologies and access to equipment, broadband and the internet” [6]. Accordingly, as early as the 2005 World Health Assembly, the WHO has urged countries to draw up long-term strategic plans for incorporating digital health in a manner appropriate for each state’s health priorities and needs [20]. At the national level, digital exclusion has enormous economic implications. For example, in the United Kingdom, a recent report by the Good Things Foundation estimated over 11 million people in the UK lack the basic digital skills to participate meaningfully in the digital economy, which translates to over £22 billion loss of revenue directly due to digital exclusion [21]. Prior to that, Mühleisen describes digital transformation as an adaptive and rapid process and lists its economic impact on various industries, including healthcare [18]. In another report focused on LMICs, McKinsey and company highlighted how 12 large-scale digital tools were adapted for use in 8 different nations during the COVID-19 pandemic [22]. All earlier work, as far as we are aware, refer to digital technology and its various facets as “super social determinants of health” with the ability to “address all other social determinants of health” [23]. The Pan-American Health Organization defines digital inclusion as the “appropriate access, digital skills, and usability and navigability in the development of technological solutions” and proposes it as one of its 8 principles for the digital transformation of the health sector [24]. Regardless of the exact terminology, all previous work agrees on the contextualization of DDOH with respect to the broader political, societal, and economic processes that they are embedded in. Namely, differences in societal preferences, socioeconomic contexts, and political and institutional configurations will generate variations in how digital technologies are incorporated and consumed in the healthcare ecosystem [1,17].

**Fig 1:**
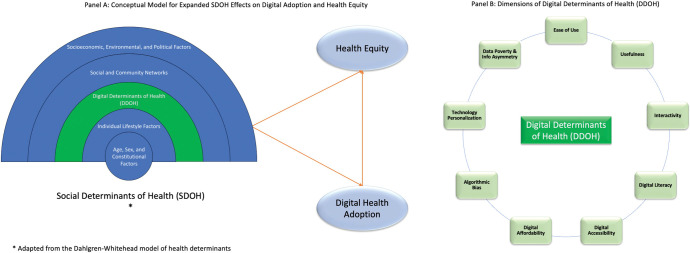
Panel A: Conceptual model for expanded SDOH effects on digital adoption and health equity. Panel B: Dimensions of DDOH.

Although factors of digital health have been studied as part of SDOH, there is little formal recognition and exploration of the field. In fact, to date, there is no widely accepted or recognized definition of DDOH [10]. Digital determinants highlight how the introduction of new technologies can influence the access and use of healthcare, and in some cases, potentiate any existing sociodemographic inequities that further impact health outcomes. In this regard, we propose a new definition of the DDOH to be used in this series of papers to achieve a common reference point. DDOH refers to the technological factors that are incorporated to provide affordable, accessible, and quality care to consumers enhancing their healthcare engagement and experience. Digital determinants refer to factors intrinsic to the technology in question that impact sociodemographic disparities, health inequities, and challenges with care accessibility, affordability, and quality outcomes (Fig 1). These include aspects such as ease of use, usefulness, interactivity, digital literacy, digital accessibility, digital availability, digital affordability, algorithmic basis, technology personalization, and data poverty and information asymmetry. Taken together, these DDOH interact closely with SDOH. Without significant empirical evidence, they can be considered as a subset of SDOH, as shown in Fig 1. Both supportive SDOH and DDOH are crucial to promote digital health adoption and health equity within populations. This calls for more empirical evidence to examine the double mediating effects of DDOH on health equity.

## Digital determinants of health and their interdependence with social determinants of health

The WHO has endorsed the need to focus on SDOH to achieve equitable and accessible care. They have clearly defined SDOH as nonmedical factors that influence health outcomes. It broadly includes the conditions in which people are born, grow, work, live, and age, and the wider set of forces and systems shaping the conditions of daily life [25]. In the Dahlgren and Whitehead model, these are categorized as individual [age, sex, and constitutional factors]; social and community networks; and general socioeconomic, cultural, and environmental conditions [e.g., education, work environment, living and working conditions, employment, water and sanitation, and healthcare services] [26]. Unlike digital determinants that concern the technology itself, social determinants relate to the external social, cultural, economic, and political factors that affect patient’s interaction with the technology. For example, a person at a lower income level may only be able to afford a version of a symptom checker with fewer functions and capabilities. While the ability to purchase an advanced version is a social determinant, the existence of different tiers of technology is a digital determinant. Within the literature, factors related to technology are often incorporated within SDOH. However, the way technology is designed, validated, used, disseminated, and incorporated within healthcare has far-reaching consequences that deserve treatment as a distinct construct. Nevertheless, both DDOH and SDOH have a closely intertwined relationship that must be considered together in their applications.

## Digital determinants of health: Digital health literacy

An individual’s health literacy is defined as the ability to find, understand, appraise, and use information and services to make health-related decisions correlates with health outcomes [27,28]. With the increasing use of digital technologies in healthcare, digital health literacy [DHL] has emerged as a high priority for healthcare organizations and governments to effectively engage consumers in their health and wellness. DHL refers to the ability of an individual to effectively interface and interact with digital technology, encompassing all the skills they require to find, understand, appraise, and apply health information specifically from electronic sources [7,9]. Previous studies have shown that an individual’s DHL influences their effective uptake of digital health [29]. Specifically, individuals with better technological skills are more informed and empowered in managing their health using digital apps, equipment, platforms, and telemedicine, which in turn is positively associated with better health-seeking, health-promoting behaviors, health knowledge, and attitudes [23]. It is, therefore, not surprising that individuals with lower DHL correlate with poorer health outcomes and typically overlap with those who are already affected by SDOH [8,30]. For example, older adults with lower educational status or income level often have a lower DHL although they are most likely to benefit from online health information. Additionally, older adults may struggle due to reduced reactivity or declining functional status or lack of motivation to learn new technology and are less likely to engage with digital health. There is also a geographical gradient to this trend, with 40% of older adults in the United States using the internet compared to 22.5% in China [31–33]. Similarly, women face structural and social barriers that hinder their participation in digital health and subsequently their literacy [34]. For example, in Uganda, men participated twice as much as women in a short messaging service [SMS]-based HIV campaign, while less than 20% of callers on a family planning hotline were women in the Democratic Republic of Congo [35,36]. Thus, women are also indirectly forced to be beneficiaries of projects without opportunities to actively engage in and shape such projects more aligned with their needs [34].

Income level determines not only accessibility to digital health but also the quality of interaction. For example, in the UK, there was a 20% difference in smartphone ownership between the highest and lowest income strata. Low-income workers were also found to be subject to practical limitations on accessing technology at work and overall had a lower understanding of how mobile phones can be used to access healthcare services [8]. In India, it is estimated that around 54 percent own smartphones. As in the case of UK, there is a difference in ownership of smartphones between the lowest and highest income groups. Even if DH sources are accessible, their applicability can be compromised due to concerns of healthcare complexity, information overload, and lack of contextualization [37]. Furthermore, technologies often have complicated interfaces that are beyond the abilities of individuals with average DHL. Thus, individuals who are most affected by health inequities tend to have a lower DHL and thus are most affected by their inability to effectively use digital technology. Understanding these factors is important to counteract the concerning trends in DHL inequity and will allow targeted interventions to be developed.

## Digital determinants of health: Telemedicine

Telemedicine has come to the forefront of healthcare, especially after the onset of the COVID-19 pandemic. It refers to traditional clinical diagnosis and monitoring that is delivered by technology [38] and includes virtual visits, remote patient monitoring, and mobile healthcare. According to Centers for Disease Control and Prevention report, over 61 million individuals in the US have a disability [39]. Despite this, telemedicine is often adept at catering to the healthcare needs of the average individual, and often is not tailored to be inclusive of patients with disabilities, who are a particularly vulnerable population with unique social, economic, and environmental disadvantages. Compared to traditional in-person healthcare, telemedicine poses several barriers to patients with physical disabilities as they are often not the intended beneficiary of design. For example, using telemedicine requires high-speed internet, but the Federal Communications Commission has reported that approximately between 21 and 42 million Americans lack high-speed internet access, and of them, physical or mental disability is a strong predictive factor for not having access to broadband internet. Furthermore, most telemedicine platforms have not been designed to cater to persons with hearing, visual, or cognitive impairments. Instead, the user interfaces are often challenging and require a keen eye for interpreting the fine print materials.

Persons with disabilities may face unique challenges specific to their type of disability. For example, effective communication over electronic formats can be difficult for individuals with intellectual disabilities, neurological, or speech disorders. Physical examination of patients with physical disabilities can be challenging if they cannot interact with the virtual interface. Patients with mental health issues or behavioral problems are often reassured by in-person physical presence, and telemedicine has not always been adapted to account for this and deliver a consultation with the same impact [40]. User interface issues such as screen reader, sign language, captions, magnification, color, contrast as well as development of novel bio-peripheral devices for physical assessments also need to be addressed [41]. These unique challenges need custom solutions to ensure that persons with disabilities are not left behind. Nevertheless, telemedicine has several advantages for patients with disabilities, including a lower transportation costs, better medication reconciliation communication, and less exposure to communicable diseases. With a tailored electronic format that considers the barriers also experienced by these groups of patients, the era of telemedicine has the potential to effect a more tangible positive impact than anticipated.

## Digital determinants of health: Artificial intelligence

Healthcare utilizes a range of data-driven technologies which work by collecting, using, and analyzing data, including patient health and care data, to support the care of individuals and patients, the functioning and improvement of health services and public health, and the development of medical research and innovation [42]. Artificial intelligence [AI] is one such data-driven technology that is its infancy for use healthcare. AI has been used in image analysis in fields such as radiology, histopathology, and dermatology [43,44]. It is the basis for many clinical decision support tools already being used in healthcare provision such as symptom checkers, patient monitors, or wearable devices. The application of AI also includes logistical support such as in automated tools that organize back-office tasks such as scheduling staff time, predicting clinic visit outcomes, and optimizing slots within clinics to reduce patients’ waiting times [45]. As with other forms of technology, AI-based works are susceptible to health inequities at every stage of the AI pipeline. Already, several reports have acknowledged the existence of biases in the design and deployment of AI technologies. For example, the accuracy of facial recognition systems from IBM is 11% to 19% less accurate in recognizing images of black men and further to 34% with images of black women [46]. This highlights not only racial but also gender-based biases within AI systems, despite the increase in ethical guidelines and standards for AI and machine learning such as the development of quality assessment of diagnostic accuracy studies for AI [QUADAS-AI] [47].

There are inherent biases within the AI technologies as a reflection of the biases ingrained within the society [48–50]. In their paper, Chen and colleagues highlighted 5 distinct stages within the AI pathway where by such biases can be understood, namely problem selection, data collection, outcome definition, algorithm development, and post-deployment considerations [51]. At the more strategic level, the healthcare priorities of minority groups are often not prioritized and hence receive less funding for AI-based solutions. For example, the funding allocated for sickle cell disease [predominantly black children affected] is 3.5 times less than that for cystic fibrosis [predominantly white children affected], albeit being a less prevalent condition [52]. If the research questions concerning disadvantaged groups are not prioritized, the structural biases will translate to less AI-based solutions as well. Given that AI outputs are dependent on the databases they are built upon, biases are possible in several ways. Firstly, the dataset itself may be underrepresented or developed based on representative data but applied to the unintended minority population [53]. Secondly, data used may have sociohistorical bias in terms of how it was entered and collected [54]. Thirdly, the data used may not account for social categories and determinants of the intended outcome. Models built on datasets with these 3 types of bias, which account for the majority, will lead to outputs that cannot be applied to patients who do not typically fit the mold and are often those already affected by health disparities.

The diffusion of innovation theory argues that individuals or countries of higher social or economic status are more likely to adopt novel technologies. In their recent MIT technology review series, Hao and colleagues emphasize that while AI can be extraordinarily useful in healthcare applications, most research has been focused on serving the more powerful populations in society. To some extent, they note examples of AI as a vehicle for the “colonization of healthcare,” whereby the outputs enrich “a powerful few by dispossessing communities that have been dispossessed before” [55]. The use of AI-based technology has several prerequisites such as decent internet access, acceptable DHL and a general understanding of how these forms of technology work and their role in the wider healthcare scene [56,57]. At the stage of deployment, patients may not be able to access these technologies or might find these technologies obsolete as it may not be directly applicable to them. This will further reduce their uptake. Some AI tools are modified based on the data input by end users, and without user data from minorities, developers may not be able to produce upgraded versions that may also cater to minority groups. Hence, there needs to be greater clarity on how to measure deployment, utilization, and patient and clinical outcomes of AI relating to ethnic equity.

## Digital determinants of health: Technologies for the atypical patient

Increasingly, organizations and governments have recognized the significant impact of digital exclusion and have initiated more efforts to reduce such disparities. For example, in the UK, the NHS long-term plan acknowledges the strong correlation between digital exclusion and individuals with characteristics that are protected under the Equality Act 2010 [58]. Consequently, the plan has made a commitment to a more concerted and systematic approach to reducing health inequities and addressing unwarranted variation in care. Besides accessibility, there is also the issue of applicability as most technologies come in a one-size-fits-all form and are not usually tailored to the specific demographic of the patient. The use of generic technologies in people it was not intended for can lead to further harm. For example, during the COVID-19 pandemic, the pulse oximeter was a significant development as it provided a noninvasive, inexpensive way to measure oxygen saturations and enable the early detection of hypoxia. However, given that pulse oximetry works by measuring the difference in light absorption between oxygenated and deoxygenated blood, the same reference intervals cannot be used for patients of different skin colors or tones. For example, Jubran and colleagues showed that while a 92% target was suitable for white Caucasian patients, a higher threshold of 95% was required to prevent significant hypoxemia in black patients. Inaccurate measurements were also twice as frequently seen in black patients than in white patients [59]. Other studies have consistently reported that different pulse oximeters have overestimated the oxygen saturations during hypoxia in darker skinned individuals [60,61]. This is especially important in a post-pandemic landscape where respiratory problems have become a more common presenting symptom. In a more recent study of 7,126 patients with COVID-19, the authors suggested that overestimation of oxygen saturation occurs frequently in racial and ethnic minority groups with that illness and leads to unrecognized or delayed recognition of eligibility to receive COVID-19 therapies [62]. The involvement of the Medicines and Healthcare products Regulatory Agency [MHRA] and the NHS Race and Health Observatory in the UK to correct this, highlight the importance of regulatory bodies in ensuring that similar technologies are more digitally inclusive.

## Digital determinants of health: Data poverty and information asymmetry

Glied and Lleras-Muney hypothesized that “improvements in health technologies tend to cause disparities in health across education groups because education enhances the ability to exploit technological advances. The most educated make the best use of this new information and adopt newer technologies first.” [63]. Health data poverty is the inability for individuals, groups, or populations to benefit from a discovery or innovation due to insufficient data that are adequately representative [64]. Health data is any information related to the physical or mental health of a person and encompasses any of the clinical, biochemical, radiological, molecular, and pathological information of a patient. Increasingly, such information is stored in an electronic format for use in future consultations. When this is carried out in a large scale, it gives rise to the amassing of large sets of health data that can be used as the basis for generating technologies. These datasets can also be used to answer research questions, inform healthcare policies, and develop new treatments. However, as with any pooled dataset, they are susceptible to biases. Key among them is the underrepresentation of minority groups as majority of the dataset will correspond with those who access it more, while neglecting those who do not use healthcare services.

Given that various technologies are developed and validated using these datasets, they are not generalizable to the wider populations, such as children, ethnic minority groups, older adults, and patients with disabilities [65]. For example, in a study aimed at predicting acute kidney injury, the model severely underperformed in female patients as only 6.4% of its initial dataset were from female patients [66]. Another example involves symptom checkers, which are built on large datasets, but these are usually not published for scrutiny and so may not necessarily incorporate minority groups [67]. Similar instances of underrepresentation have been seen in diagnosing skin lesions, as most algorithms do not include skin lesions in ethnic minorities [68]. This can both reinforce existing health inequities and cause possible harm among minority patients, giving rise to other associated ethical issues. Instead of narrowing the health gap, such technologies widen the digital divide through the health data poverty borne out of asymmetrical datasets.

## Policy implications and future work: Where do we go from here?

Based on the above discussion, there are many and significant implications of using digital health technology. These can be analyzed in terms of the stakeholders involved, namely the individual patient, developers of digital technology, service providers [hospitals and physicians in both public and private sectors], and government bodies. Future work must take into consideration these implications within the broader context of social, demographic, and economic profiles of each individual; the healthcare structure they are embedded in; and the healthcare priorities of the broader community.

### Individuals

At the individual level, patients and public need to be more aware of the digital transformation of health services. The COVID-19 pandemic has unpredictably ushered and in some cases forced patients and providers to adopt digital health. These experiences have also highlighted that not all factions of a population and care providers are prepared to use digital health, specifically if it becomes the only mechanism to deliver care. Consequently, there is resistance to adopt digital health until trust is established in regards to its reliability, quality, ease of use, and usefulness (Fig 1). Individuals need to develop a better awareness of their limitations, appreciate the benefits, and be motivated to acquire new skills that will enable them to use digital technology. The physician–patient relationship, in most instances, is already not an equitable relationship given that the physician and patient have large differences in healthcare expertise and access to specific details that create information asymmetry and consequently influence decision-making. The premature adoption of technology can potentially exacerbate this imbalance in an era where shared decision-making is actively encouraged. Furthermore, there are several government initiatives and programs aimed at improving individuals’ DHL and obtaining their views on potential digital technologies through patient and public involvement and engagement [PPIE] schemes. Patients can participate in these programs to ensure their views and profiles are incorporated into relevant policy-making exercises.

### Developers

Digital health technology takes various forms, including AI-based tools, telemedicine, symptom checkers, mobile phone applications, precision medicine, and robotic technology. While profit margins, revenue and return on investments are key factors in the design and development of these technologies, in the future era developers need to consider their social impact as well [69]. The importance of digital inclusion must become a core principle at the outset of technology design and not an afterthought as it currently is with many health tools. For example, as of 2021, there are approximately 3 million applications on Google Play store alone, with health and fitness being the biggest category [70]. Although this figure is expected to rise further, the majority of the world population still does not have access to either a smartphone or high-speed internet to access these apps. To achieve this, developers can conduct market research to better understand the socioeconomic, demographic, and political profiles of their end users, and finetune their technology accordingly. For example, the same presenting symptoms will yield a different set of possible diagnoses in different countries, so symptom checkers can map out the epidemiological differences of medical conditions between different countries and modify their algorithms to account for local trends [71]. Within most randomized clinical trials, there is now a greater requirement for PPIE. The same approach can be extended to technology development, whereby developers incorporate the views and needs of their end users to ensure the production of an inclusive technology [72,73].

### Service providers: Partnerships in public and private sectors

Service providers serve as the bridge between developers, government organizations, and the end users [patients and the public]. Hospitals have become more open to using technology for delivering healthcare in recent years, but they have not necessarily been equipped with the necessary infrastructure. In the post-pandemic era, physicians have adopted digital health but may not be cognizant of their own biases and limitations. For example, not many physicians may have been trained to carry out clinic consultations in a virtual format prior to the COVID-19 pandemic [74]. When patients with physical disabilities, visual or hearing impairments are involved, the challenges multiply and can reduce the effectiveness of the health episode. Accordingly, telehealth medicine training is being incorporated into current training systems [75,76].

Compared to previous healthcare interventions, the boundaries between private and public healthcare players become more blurred. In the digital health market, there is a significant interaction between service providers and private companies. For example, over 184 venture capitalist investments were made by service providers in 105 companies over the 2011 to 2019 period [69]. All major technology companies, including Alibaba, Alphabet, Amazon, Apple, Facebook, Jio, Microsoft, and TenCent are now expanding their reach into the health sector. Significant examples include Amazon Care and the TenCent Smart Hospital, where the former was formally recruited by the Chinese government as part of the national AI strategy [77,78]. Telecommunication networks will need to buy into the digital strategy to support with necessary information and communications technology [ICT] software and hardware. While such partnerships can foster better transfer of information and ideas, it also belies the risks of data privatization of extreme privatization of health data and exacerbate existing health inequities. In a corporate environment motivated by economic gains, service providers must ensure that the patient is still prioritized and placed at the center of healthcare provision.

### Government bodies and organizations

Governments need to employ an equity and rights-centered approach towards digital health and give importance to the needs of people with least power and most dependent on the government, including children, youth, elderly, women, people with disabilities, and other minority and marginalized factions of society [79–81]. This can be achieved by ensuring that individuals are “digital health ready.” Digital health readiness refers to the variable extent to which individuals and countries have the capacity to use digital technology and data for improving their own or their population’s health and wellbeing [1]. Governments can identify individuals who lack digital health readiness and implement policies that directly alleviate it. Simultaneously, governments can partner with service providers, developers, and companies to ensure that digital health technology produced is ethical and equitable by including the needs and sentiments of minority groups [82].

Within countries, there are dedicated public organizations which are responsible for approval of digital technologies, such as the MHRA in the UK, Food and Drug Administration [FDA] in the USA, Central Drugs Standard Control Organisation in India, and National Medical Products Administration in China. These organizations can play a bigger role in monitoring health technologies. Within the UK, there is already an increased awareness of digital health inequities and has led to initiatives such as increased funding solely dedicated to resolving them [45,83]. Currently, there is a specific emphasis by regulatory bodies on the safety and effectiveness of digital tools. Future priorities of regulation must be an equally strong focus on health technology being equitable and inclusive. Regulatory bodies can also play a greater role in the concomitant monitoring and reevaluation of any digital technology approved for use. Ultimately, stronger and more collaborative digital relationships between countries and networks such as the European Union or the OECD need to be forged to allow for smooth transition and sharing of newer technologies, data, and ideas and provide development assistance for digital health in LMICs. To date, the US Agency for International Development has published its plans in a report entitled Vision for Action in Digital Health. Within economic and geopolitical networks, a more coordinated plan is necessary to elevate the overall introduction, implementation, and evaluation of digital technologies globally.

There is global variation in the uptake of digital health. As per the WHO global strategy on digital health, more than 120 member nations have adopted such policies, but this implies the lack of similar strategies in the remaining countries [6]. Furthermore, the effectiveness of these policies is variable, questionable and unknown in most settings. Of course, there is a diverse range of work carried out confirming the effectiveness of digital technology in developed countries, but these studies also characterize a variation in the demographic reach even in high-income countries, with the lower socioeconomic cohorts inevitably left behind. If this is the case in wealthier nations, the variation in the reach of digital health technologies and the respective policies is doubtful and alarming. For example, the 99DOTS program is a mobile phone-based initiative for monitoring tuberculosis medication adherence among more than 150,000 patients in India’s public health sector. Although hailed as a successful public and digital health initiative, studies have reported poor medication adherence and premature cessation of therapy due to poor cell phone accessibility, cellular signal, and literacy [15]. Similarly, the COVID-19 pandemic led to increased and accelerated global uptake of telemedicine, symptom checkers, and mobile phone applications [84]. While success stories have been reported in wealthier nations, similar reports are more scarce in LMICs [85,86]. Lastly, the political systems of different countries introduce another complexity to how digital health can be prioritized on the agenda [87,88]. Overall, even if there is a widespread digital health strategy present, its practical implementation is open to challenges, and governments need to constantly introduce, evaluate, and adapt their digital health policies.

## Conclusion

With the increasing use of digital health in healthcare, the potential for health inequities it poses must be addressed. In tandem with ongoing work to minimize the digital divide cause by existing SDOH, further work is necessary to recognize digital determinants as an important and distinct entity. This will allow for dedicated efforts to address their impact and lobby organizations, regulatory bodies, health systems, and governments to design technology that is truly digitally inclusive. In the remainder of the series, we will evaluate on each of the subtopic outlined in this introductory topic and expand upon their impact and implications on delivery of healthcare services in a safe and equitable manner.
